# Supplementary material for: Bayesian refinement of protein structures and ensembles against SAXS data using molecular dynamics
Source: PLoS Comput Biol. 2017 Oct 18;13(10):e1005800. doi: 10.1371/journal.pcbi.1005800 (PMC5662244; doi:10.1371/journal.pcbi.1005800)
Supplement: S1 Archive — (BZ2) [file pcbi.1005800.s011.tar.bz2 › gromacs_4.62_bayesian_saxs_refi/share/html/online/g_mdmat.html]

g\_mdmat


| g\_mdmat Main Table of Contents |

**VERSION 4.6  
Sat 19 Jan 2013**

---

### Description

g\_mdmat makes distance matrices consisting of the smallest distance
between residue pairs. With -frames, these distance matrices can be
stored in order to see differences in tertiary structure as a
function of time. If you choose your options unwisely, this may generate
a large output file. By default, only an averaged matrix over the whole
trajectory is output.
Also a count of the number of different atomic contacts between
residues over the whole trajectory can be made.
The output can be processed with xpm2ps to make a PostScript (tm) plot.

### Files

| option | filename | type | description |
| --- | --- | --- | --- |
| **-f** | traj.xtc | Input | Trajectory: xtc trr trj gro g96 pdb cpt |
| **-s** | topol.tpr | Input | Structure+mass(db): tpr tpb tpa gro g96 pdb |
| **-n** | index.ndx | Input, Opt. | Index file |
| **-mean** | dm.xpm | Output | X PixMap compatible matrix file |
| **-frames** | dmf.xpm | Output, Opt. | X PixMap compatible matrix file |
| **-no** | num.xvg | Output, Opt. | xvgr/xmgr file |

### Other options

| option | type | default | description |
| --- | --- | --- | --- |
| **-[no]h** | bool | no | Print help info and quit || **-[no]version** | bool | no | Print version info and quit || **-nice** | int | 19 | Set the nicelevel || **-b** | time | 0 | First frame (ps) to read from trajectory || **-e** | time | 0 | Last frame (ps) to read from trajectory || **-dt** | time | 0 | Only use frame when t MOD dt = first time (ps) || **-xvg** | enum | xmgrace | xvg plot formatting: xmgrace, xmgr or none || **-t** | real | 1.5 | trunc distance || **-nlevels** | int | 40 | Discretize distance in this number of levels |

---

http://www.gromacs.org  
gromacs@gromacs.org
